# Question Order Effects in Multidimensional Risk Perception Measurement

**DOI:** 10.1111/risa.70164

**Published:** 2025-12-16

**Authors:** Savannah J. Meier, Hwanseok Song

**Affiliations:** ^1^ Brian Lamb School of Communication Purdue University West Lafayette Indiana USA

**Keywords:** analytic thinking, numeracy, order effects, risk perception

## Abstract

This study examines how question order influences responses in multidimensional risk perception measurement. Through a randomized between‐subjects experiment (*N *= 1352) manipulating the sequence of risk perception dimensions, we identified systematic question order effects. When a general risk question followed specific dimensional questions, responses showed significant assimilation effects (i.e., general risk aligned more closely with preceding specific dimension ratings). Consequence dimension responses (severity, affect) showed assimilation effects when preceded by probability dimensions (exposure, susceptibility), while probability dimensions remained stable regardless of ordering. Within subdimensions, severity ratings were influenced by preceding affect questions, and susceptibility ratings were influenced by preceding exposure questions, both displaying assimilation patterns. Testing how individual differences in cognitive sophistication moderate susceptibility to order effects, contrary to our predictions, we found that individuals higher in analytical thinking style demonstrated stronger order effects for general risk questions than those lower in analytical thinking. These findings reveal an asymmetrical pattern where judgments requiring more analytic specificity tend to anchor evaluations that are relatively global, affective, or self‐focused.

## 1 Introduction

Early research on risk perception illuminated the ways in which lay perceptions of risk systematically differ from technical risk assessments (Fischhoff et al. 1978; Slovic 1987), spurring decades of research revealing how characteristics of hazards, as evaluated by individuals, influence behavior and decisions related to risk (Siegrist and Árvai 2020). Subsequent research further demonstrated that although experts typically focus on quantifiable harms and benefits as well as exposure probabilities in their risk assessments, lay perceptions of risk are also shaped by qualitative hazard characteristics (McDaniels et al. 1997; Weber et al. 2001; White et al. 2004) as well as other heuristics such as trust (Siegrist 2021) and affect (Finucane, Alhakami, et al. 2000; Slovic et al. 2002). A general consensus emerged among researchers that risk perception could be conceptualized as an amalgam consisting of both deliberative and emotional components (Slovic et al. 2004).

Nevertheless, at the operational level, development of instruments comprehensively capturing these alternative dimensions of risk perception remains an evolving endeavor. Whereas many traditional measures of risk focused on deliberative dimensions of risk (e.g., perceived likelihood), neglecting the role of affect, other measures have operationalized risk perception with exclusively affective (e.g., concern, worry, fear) measures (Wilson et al. 2019). More recently, scholars have developed, validated, and refined scales that measure risk perception more comprehensively, accounting for both deliberative and affective elements of the construct (Ferrer et al. 2016; Walpole and Wilson 2021a, 2021b; Wilson et al. 2019).

While these studies have primarily focused on developing the content of risk perception scales that validly reflect the construct, we also believe that their multidimensional nature gives rise to new contextual effects that warrant careful consideration. That is, individuals’ responses to alternative dimensions of risk perception may be influenced by the order in which the dimensions are asked—a topic that has received little attention in the field. For example, do responses to a general question about a hazard's perceived risk differ when it is answered after specific questions about perceived probability or consequences? Moreover, do these question order effects manifest differently across individuals with varying levels of cognitive predispositions and ability?

To answer these questions, we built on recent research on multidimensional risk perception scales (Walpole and Wilson 2021a; Wilson et al. 2019) and conducted a randomized between‐subjects experiment manipulating the order of different dimensions and subdimensions of risk perception scales. We first present a general overview of recent work on multidimensional risk perception scales followed by a literature review of question order effects and potential individual differences that may moderate these processes.

## 2 Literature Review

### 2.1 Conceptualizing and Operationalizing Risk Perception

Across diverse definitions, risk is typically conceptualized as a function of negative consequences, which vary in levels of severity, and uncertainty, which is characterized as the probability of the consequences’ occurrence (Aven et al. 2018). Early conceptualizations of risk perception often mirrored these elements of risk that are often central to technical risk assessments, treating risk perception as a cognitive construct comprising individual beliefs about severity and susceptibility. For example, the Health Belief Model (Janz and Becker 1984; Rosenstock 1966) posited that perceived seriousness of and susceptibility to a disease were two main components of perceived threat. It is worth noting that Rosenstock (1966) explicitly acknowledged the role of emotional arousal in perceived seriousness. However, subsequent theorization that evolved into the Protection Motivation Theory (Rogers 1975; Rogers and Mewborn 1976) and the Extended Parallel Process Model (Witte 1992) tended to consider perceptions of severity and susceptibility as more deliberative constructs (i.e., perceived threat) distinct from affective states such as fear. Other research programs such as unrealistic optimism (Weinstein 1980, 1987) showed an even heavier cognitive focus, equating risk judgments exclusively with perceptions of event probabilities.

Early risk perception research based on the psychometric paradigm revealed that individuals’ perceptions of risk extend beyond deliberative judgments central to technical risk assessments. Slovic (1987) demonstrated that risk perception arises from perceived hazard characteristics, which could be summarized into two primary dimensions: the level of dread associated with the hazard and the extent to which the hazard was perceived as unknown. In particular, the dread dimension, which represented affective and visceral reactions to a hazard (e.g., controllability, catastrophic potential, voluntariness, equitability), emerged as more influential than the unknown dimension in predicting global risk perception and regulatory support. Similarly, Sandman (1993) used the term “outrage” to broadly refer to public concerns associated with a risk that are distinguished from magnitude and probability assessments (i.e., *hazard*), which tend to be the focus of experts.

Later theorization further explicitly acknowledged the role of affect as a core component of risk perceptions alongside deliberative assessments. Loewenstein et al. (2001) proposed a “risk‐as‐feeling” hypothesis, which suggested that emotions such as worry, fear, dread, and anxiety and deliberative evaluations such as event probability or consequence desirability mutually influence each other. These researchers also argued that emotions can be directly influenced by hazard characteristics (e.g., immediacy of risk) and subsequently guide judgments and decisions without mediation of cognitive assessments. Similarly, research on the affect heuristic revealed that instead of judging risks and benefits of various activities and technologies independently, individuals may be motivated to report these judgments in a way consistent with their subtle feelings of positivity or negativity associated with the hazard (Finucane, Alhakami, et al. 2000; Slovic et al. 2002).

More recently, noticing that most risk perception measures fail to consider the deliberative and affective dimensions together, researchers have developed multidimensional scales to improve the construct's operationalization. Ferrer et al. (2016) proposed a tripartite model of risk perception in which disease risk is measured using three dimensions: deliberative, affective, and experiential. Although items in their deliberative dimension focused on the likelihood of contracting a disease, their affective dimension measured discrete emotions in response to the disease such as worry, fear, and anxiety. The experiential dimension measured gut‐level reactions concerning vulnerability to the disease. Similarly, following a review of risk perception measures in the literature, Wilson et al. (2019) proposed a scale consisting of three dimensions—affect, probability, and consequence—that was designed to be applicable to a broad range of hazard contexts. Their confirmatory factor analysis found support for this multidimensional structure across four hazard types, although the correlation between each dimension and a general measure of risk perception (i.e., How risky is X?) varied across the hazard types in multiple regression models. Further building on this work, these researchers proposed and tested an enhanced scale with a four‐dimensional structure that separated the original probability dimension into exposure (likelihood of experiencing a hazard) and susceptibility (likelihood of suffering a negative consequence, given exposure to a hazard) (Walpole and Wilson 2021a, 2021b). This four‐factor structure was validated, explaining additional variance in general risk beyond the three‐factor structure in Wilson et al. (2019). The two subdimensions pertaining to perceived consequences of the hazard (i.e., affect, severity) consistently predicted general risk across hazard types. In contrast, the two probability subdimensions (i.e., exposure, susceptibility) correlated with general risk less consistently across hazard types and less strongly than did the consequence subdimensions. Following refinement of the scale using item response theory modeling, Walpole and Wilson (2021a) presented an economic 13‐item scale representing the four dimensions of risk perceptions (i.e., consequence: affect and severity; probability: exposure and susceptibility).

### 2.2 Order Effects Across Risk Perception Dimensions

We argue that in addition to the content of the scale items, the order in which individuals answer these questions needs to be considered. That is, question order effects in which preceding questions systematically influence responses to questions that follow (Strack 1992) may occur when survey participants answer questions about certain risk dimensions before others. An extensive body of research suggests that within the multidimensional scale developed by Walpole and Wilson (2021a), question order may systematically influence how people respond to general versus specific risk perception questions and consequence versus probability dimension questions.

#### 2.2.1. Order Effects of General vs. Specific Questions

In general, question order effects may manifest in one of two patterns. An assimilation effect occurs when earlier questions influence responses to later questions to become more similar. On the other hand, contrast effects occur when preceding questions influence responses to later questions such that responses to the two sets of questions become more divergent (Schuman and Presser 1981).

Research shows that in surveys asking both general and specific questions, specific questions remain relatively stable, whereas general questions are influenced by preceding specific questions (Bjarnason and Jonsson 2005; Lee et al. 2023). Driven by alternative processes, both assimilation and contrast effects have been reported. For example, Schuman et al. (1981) found a contrast effect on general questions in which support for the general question about legal abortion was lower when it followed a specific question asking whether one supports legal abortion if there is a strong chance of a serious defect in the baby. Similarly, survey respondents reported lower levels of happiness in general when they answered a question about their marital happiness, which was typically high, first (Schuman and Presser 1981). Explaining these contrast effects, Schuman and Presser (1981) surmised that respondents assume that a general question following a specific question implicitly requests the respondent to exclude the previous specific example in formulating their answer (cf. Stark et al. 2020). However, subsequent research conducted in different contexts frequently found assimilation effects. Smith (1982) found that those who were happy about their marriage reported greater levels of general happiness when this question followed the specific question about marital happiness. In the health domain, Garbarski et al. (2015) similarly found an assimilation effect in which correlations between a general self‐rated health item and domain‐specific health items increased when the specific questions were asked before the general question. Assimilation effects can be explained with simple priming—when individuals answer specific questions first, the recalled information shapes answers to the general question that follows (Lee et al. 2023).

#### 2.2.2. Assimilation vs. Contrast in General Questions

To explain when assimilation versus contrast effects manifest in general questions following specific questions, Schwarz et al. (1991) proposed and tested a model based on conversational logic. According to these researchers, when a general question follows one specific question belonging to the same conversational context, responses will be influenced by conversational norms that prohibit redundant use of the same information, resulting in a contrast effect. However, when the general question follows one specific question perceived to be appearing in a different conversational context, informational priming will shape responses to the general question, resulting in an assimilation effect. In addition, when a general question follows *multiple* specific questions, it will always be treated as a request for a summary judgment, resulting in an assimilation effect even when the general and specific questions belong to the same conversational context. Their experiment, which manipulated both the number of specific questions and whether the general and specific questions appeared in the same context, provided support for this model. Subsequent research similarly found support for the summarizing nature of general questions following multiple specific questions (Bless and Schwarz 2010; Kaplan et al. 2013; Thau et al. 2021).

Because the risk perception scale developed by Walpole and Wilson (2021a) consists of one general risk item and multiple specific questions measuring four dimensions of risk perception, we believe the question order effect literature discussed here is particularly pertinent. Given the literature showing assimilation effects when a general question follows several specific questions, we predict the following:

H 1 When the general risk perception question follows specific risk perception questions, responses will show assimilation toward the specific questions as measured in the specific‐first condition (where specific questions are uninfluenced by order effects). That is, the general risk rating will shift toward the unconfounded mean of specific dimensions compared to when the general question is asked first.

In addition, considering how answers to specific questions remain relatively stable regardless of whether they are preceded by general questions or not (Bjarnason and Jonsson 2005; Lee et al. 2023), we predict the following:

H 2 The position of the general risk perception question (first vs. last) will not affect responses to the specific risk perception questions.

#### 2.2.3. Order Effects of Consequence vs. Probability Dimensions

In addition to order effects on general questions, order effects may occur between specific risk perception dimensions. Theorization of affect in risk research has demonstrated that emotions are more closely related to consequences than probability (Loewenstein et al. 2001; Slovic et al. 2002). This distinction is also reflected in contemporary risk perception measures (Walpole and Wilson 2021a), which suggest that the consequence dimension (i.e., affect, severity) tends to represent more affective appraisals of the hazard, whereas the probability dimension (i.e., exposure, susceptibility) tends to represent more deliberative appraisals.

Thus, the psychological literature on how emotional states and cognition interact through priming processes can help illuminate potential order effects between consequence and probability dimensions. First, consequence questions may influence responses to subsequent probability questions by eliciting either pleasant or unpleasant emotional states. In an early study on this topic, Schwarz and Clore (1983) found that participants rated their life satisfaction more positively when asked on a sunny day (positive mood) than on a rainy day (negative mood). Interestingly, this effect disappeared when participants were sensitized to the possibility that mood could influence their responses to the question. This foundational study was later expanded into a “feelings as information” model, which holds that individuals use feelings as a source of information because their relevance is rarely questioned and accordingly accepted by default (Schwarz 2012). For example, Moy et al. (2001) found that priming participants with affect by asking them about their attitudes toward the death penalty first resulted in greater levels of affective content in their subsequent thought‐listing task. Sweitzer and Shulman (2018) found that positive versus negative metacognitive experiences, induced by manipulating the questions’ vocabulary level difficulty in a public opinion survey, altered how participants rated their own interest and efficacy in politics. These findings suggest that responding to the consequence dimensions (affect, severity) first may prime individuals with affective states that subsequently color more deliberative probability judgments. Thus, we hypothesize the following:

H 3 When the probability dimension follows consequence dimension questions, responses will show assimilation toward the consequence dimension as measured in the consequence‐first condition (where consequence questions are uninfluenced by order effects). That is, the probability dimension will shift toward the unconfounded mean of the consequence dimension compared to when the probability dimension is asked first.

Conversely, the deliberative probability assessments may anchor subsequent consequence evaluations by increasing the cognitive accessibility of statistical information. More deliberative questions about risk perception pertaining to exposure and personal susceptibility may lead individuals to integrate these elements when they formulate a response asking about the hazard's severity or feelings toward the hazard. In this process, priming would increase the cognitive accessibility of a specific belief, increasing the likelihood that this belief would be used in later affective evaluations of the stimulus. Supporting this accessibility account, McClendon and O'Brien (1988) found that in order effects where answering satisfaction with specific life domains (e.g., marriage, health, employment) affected a general well‐being question, domains that were located more closely before the general question displayed greater assimilation effects. Similarly, Bowling and Windsor (2008) found that participants reported their health status overall more positively after they answered questions about various health issues. It is likely that the specific health items brought their attention to both positive and negative aspects of their health holistically, whereas those who answered the general question first were focusing more on the health problems they had because of their higher accessibility in memory. Moy et al. (2001) also found that those who were asked to list their cognitions about the death penalty first showed high levels of consistency between their cognitions and their affective summary of their position (i.e., attitude). Applied to multidimensional risk perception, this accessibility mechanism suggests that when probability dimensions precede consequence dimensions, the cognitive accessibility of deliberative risk information should systematically influence subsequent affective evaluations. This reasoning aligns with our earlier discussion of how consideration of specific features of a hazard may systematically anchor more global evaluations in the general‐specific order effect. Extending this accessibility account, we predict the following:

H 4 When the consequence dimension follows probability dimension questions, responses will show assimilation toward the probability dimension as measured in the probability‐first condition (where probability questions are uninfluenced by order effects). That is, responses to the consequence dimension will shift toward the unconfounded mean of the probability dimension compared to when the consequence dimension is asked first.

#### 2.2.4. Order Effects Among Subdimensions of Consequence and Probability

On the other hand, relatively scant research is available to predict order effects among subdimensions under consequence (severity vs. affect first) and probability (exposure vs. susceptibility first) dimensions, likely because these specific distinctions are unique to risk research (rather than broader public opinion or psychological literature) and multidimensional risk perception scales (which are fairly novel developments). Nevertheless, addressing this gap is important because as other question order effects described above, subdimension order effects could introduce systematic measurement artifacts.

Related to potential order effects between the two subdimensions of probability (i.e., exposure and susceptibility), Gold and Barclay (2006) conducted an order effects experiment situated in the context of unrealistic optimism where they asked a direct‐comparison question (i.e., own likelihood of experiencing a negative event compared to the average same university student) versus a self‐risk question in alternative sequences. They found that when the direct‐comparison question was asked first, the correlation between the two items increased. However, Walpole and Wilson's (2021a) exposure and susceptibility measures are distinct from these items in that their exposure subdimension measure asks about community‐level occurrence of a negative event, whereas the susceptibility dimension measures the likelihood of experiencing negative effects provided such exposure, without requiring a comparison between self and community susceptibility. Given the lack of research in this area, we ask the following:

RQ1: Are there order effects between severity and affect subdimensions of risk perception?
RQ2: Are there order effects between exposure and susceptibility subdimensions of risk perception?

### 2.3 Individual Differences as Moderators of Order Effects

Additionally, we surmised that whenever order effects are observed, individual differences in motivation and ability to engage in cognitive tasks would moderate such order effects. Prior research suggests that order effects tend to be more pronounced among those with lower educational attainment because they are more likely to find the survey cognitively demanding and thus become more susceptible to contextual influences as a less effortful way to provide their responses (Lee et al. 2023; Narayan and Krosnick 1996; Schuman and Ludwig 1983). If cognitive sophistication is indeed a key moderator of question order effects, scales assessing motivation and ability to engage in analytical thinking can serve as more direct moderators in these cognitive processes. The Rational‐Experiential Inventory (Epstein et al. 1996) uses two subscales, need for cognition (Cacioppo and Petty 1982) and faith in intuition, to independently measure individual differences in analytical (rational) versus intuitive (experiential) thinking styles, respectively. For example, Song and Schuldt (2017) found that individuals with high scores in rationality evaluated risks of threatened species more consistently, being less influenced by differences in affect evoked across alternative statistical criteria. As for cognitive ability, more numerate individuals have been found to be less influenced by equivalency framing effects (Peters et al. 2006) and irrelevant anchors (Helm et al. 2020), providing more logically consistent responses where they are normatively expected than the less numerate. Similarly, individuals higher in cognitive reflection, a tendency to override an intuitive but incorrect first response and engage in further reflection to pursue the correct answer (Frederick 2005), tend to show greater consistency between reversed and direct questions in surveys (Fukudome and Takeda 2024). Accordingly, we test whether these individual differences moderate our hypothesized question order effects, predicting that in general, greater cognitive sophistication will reduce order effects.

H 5 Across identified order effects, individuals lower in (a) education attainment, (b) analytical (rational) thinking style, and (c) cognitive ability will exhibit greater question order effects. However, (d) individuals higher in intuitive (experiential) thinking style will exhibit greater question order effects.

## 3 Method

We conducted an online experiment with four different order manipulations, two levels each, resulting in a 2 (general‐first vs. specific‐first) × 2 (within specific questions: consequence‐first vs. probability‐first) × 2 (within the consequence dimension: severity‐first vs. affect‐first) × 2 (within the probability dimension: exposure‐first vs. susceptibility‐first) between‐subjects design. Participants were randomly assigned to one of the 16 conditions, each of which presented questions in a different order. Regardless of the order, subdimensions of consequence (severity and affect) were always paired together without any probability questions (susceptibility and exposure) inserted between and vice versa. For example, a participant who was assigned to the specific‐first–probability‐first–severity‐first–susceptibility‐first condition would have responded to question blocks in the order of susceptibility, exposure, severity, affect, and general risk.

### 3.1 Participants

We recruited 1352 adult US residents from MTurk via CloudResearch, applying the latter platform's filter for participants known to provide high‐quality responses. An a priori sample size analysis performed using G*Power (Faul et al. 2009) indicated that a minimum of 1302 participants would be required to detect a small effect size (*f* = 0.10) with Type I and Type II error rates of 0.05, respectively. Our final sample was balanced in gender distribution (48.9% female). The median age group was 35–44 years old. About 62.1% reported having a bachelor's degree or higher. About 6.3% identified as Hispanic or Latino, while 80.5% identified as White and 10% as Black or African American. In terms of political ideology, the sample leaned liberal, with 44.4% identifying as liberal or very liberal (27.4% conservative or very conservative).

### 3.2 Procedure

Upon completion of the form to indicate informed consent, participants were randomly assigned to a question order experimental condition. They were also randomly assigned one of four hazard items for which they rated their risk perception. These hazard items were identical to those used by Walpole and Wilson (2021a): tornadoes, lead contamination, heatwaves, and poor air quality days. Because we had no theoretical interest in order effect differences across hazards, we treated hazard type as a control variable in our analyses. Participants first completed the multidimensional risk perception scale (Walpole and Wilson 2021a) with dimensions ordered according to their experimental condition. Next, they answered scales measuring cognitive sophistication including the Rational‐Experiential Inventory (Epstein et al. 1996) and the Rasch‐based numeracy scale (Weller et al. 2013). Finally, participants responded to demographic questions.

### 3.3 Measurement

#### 3.3.1 Multidimensional Risk Perception

To measure multidimensional risk perception, we used the scale developed by Walpole and Wilson (2021a). This scale consists of 13 items with four subscales measuring the subdimensions of severity, affect, exposure, and susceptibility (Table 1). Of these, severity and affect comprise the consequence dimension, whereas exposure and susceptibility comprise the probability dimension. All items were measured using 5‐point Likert‐type scales. All four subscales were reliable, *α*_severity_ = 0.81, *α*_affect_ = 0.92, *α*_exposure_ = 0.90, and *α*_susceptibility_ = 0.94. To test the order effects between general and specific items, we also adopted a general risk item from Wilson et al. (2019), a study that featured an earlier version of the multidimensional scale we used.

**TABLE 1:** Multidimensional risk perception dimensions and items.

Category	Item
General	How risky is [hazard]?
Consequence	
Severity	How severe would you expect the consequences of [hazard] to be?
	How severe would the impacts of [hazard] be to you if you were to suffer them?
	If you were to suffer the consequences of [hazard], how severe would they be likely to be?
Affect	How concerned are you, if at all, about [hazard]?
	When you think about [hazard] for a moment, to what extent do you feel worried?
	How afraid are you, if at all, of [hazard]?
Probability	
Exposure	How likely is it that [hazard] will occur in your community?
	How often do [hazard] occur in your community?
	To what extent do you feel you might experience [hazard] in your community?
	How easy or difficult is it to imagine [hazard] occurring in your community?
Susceptibility	If you did experience [hazard] in your community, how likely is it that it would negatively impact you?
	If [hazard] were to occur in your community, how vulnerable would you be to the impacts?
	If [hazard] occurred in your community, how likely would you be to suffer consequences?

*Note*: All items are adopted from Walpole and Wilson (2021a) except for the general risk question adopted from Wilson et al. (2019).

#### 3.3.2 Individual Differences in Cognitive Sophistication

Educational attainment was measured as a demographic variable. To assess individual thinking style, we used the Rational‐Experiential Inventory by Epstein et al. (1996). Each of the two subscales, rationality and experientiality, consisted of five items, with three items in the rationality subscale reverse‐coded. All items were measured on a 5‐point Likert‐type scale. Both subscales were reliable, *α*_rationality_ = 0.86 and *α*_experientiality_ = 0.92. To assess cognitive ability, we used the Rasch‐based abbreviated numeracy scale developed by Weller et al. (2013), which combines items from objective numeracy scales (Lipkus et al. 2001; Peters et al. 2006) and the Cognitive Reflection Test (Frederick 2005).

### 3.4 Data Analysis

We used a two‐step process to determine the occurrence and direction of order effects. In the first step, for each hypothesis, we tested the presence of the order effect using general linear models (UNIANOVA procedure in SPSS 30) with the relevant order manipulation entered as the independent variable, hazard type as a control variable, and the target risk perception dimension as the dependent variable.

For any significant order effect identified in the first step, we then determined whether it represented an assimilation or contrast effect using two complementary approaches: (a) comparing the means of a target dimension between two alternative order conditions and (b) analyzing how the relationship between the target and anchor dimensions changes depending on alternative question orders (Lee et al. 2023). First, we determined that an assimilation or contrast effect occurred when means between the dimensions converged or diverged when presented in a particular order. For example, it was judged that an assimilation effect occurred on a target dimension (e.g., consequence) if the mean response to the target dimension moved toward the unconfounded mean of the anchor dimension (e.g., probability, as measured in the probability‐first condition) when the target dimension was answered after the anchor dimension (i.e., probability‐first condition) rather than before (i.e., consequence‐first condition).

As a secondary measure to discern assimilation versus contrast effects, we tested whether the relationship between the target and anchor dimensions strengthened or weakened when presented in a particular order. We used the general linear model (UNIANOVA) procedure in SPSS, entering the mean score of the anchor dimension as the independent variable, the order effect variable as a moderator, and hazard type as a covariate in the model, predicting the score of the target dimension. If the anchor dimension × order interaction effect emerged as significant, its coefficient from parameter estimates was interpreted to determine whether the effect indicated assimilation or contrast.

## 4 Results

Table 2 displays the mean of each dimension depending on the relevant question order manipulation. H1 predicted an assimilation effect on the general risk perception question when it is answered after specific risk perception questions. General risk perception was significantly different between the general‐first and specific‐first conditions, *F*(1,1347) = 45.44, *p* < 0.001, 𝜂_p_^2^ = 0.033. As predicted, general risk perception ratings converged with the unconfounded mean of specific questions (i.e., the mean of specific questions in the specific‐first condition), *M* = 3.05, SD = 0.79, when it was asked after specific questions, *M* = 3.74, SD = 1.08, rather than before, *M* = 4.08, SD = 1.04. Further supporting this assimilation trend, the correlation between the means of general and specific questions was stronger in the specific‐first condition, as indicated by the positive interaction effect, *F*(1, 1345) = 4.18, *p* = 0.041, *η_p_* ^2^ = 0.003. Specifically, the coefficient of specific questions predicting general questions was 0.61 in the general‐first condition but increased to 0.72 in the specific‐first condition. H1 was supported.

**TABLE 2:** Descriptive statistics of each dimension by question order.

Dimension	Order: (A)→(B)			Order: (B)→(A)		
	*n*	*M*	SD	*n*	*M*	SD
General (A)	679	4.08^a^	1.04	673	3.74^b^	1.08
Specific (B)		3.11^a^	0.84		3.05^a^	0.79
Consequence (A)	669	3.29^a^	0.99	683	2.98^b^	1.05
Probability (B)		3.07^a^	0.81		3.07^a^	0.75
Severity (A)	653	3.58^a^	1.15	699	3.42^b^	1.15
Affect (B)		2.75^a^	1.20		2.79^a^	1.21
Susceptibility (A)	675	3.47^a^	1.08	677	3.26^a^	1.12
Exposure (B)		2.72^a^	1.03		2.83^b^	1.04

*Note*: In each pair of dimensions tested for order effects, one dimension is labeled (A) and the other (B). Means in the same row not sharing a superscript are significantly different from each other at *p* < 0.05 (two‐sided) according to independent samples *t*‐tests. Means for each dimension by hazard type and question order can be found in the Supporting Information (Table S1).

In contrast, H2 predicted that the specific risk questions would not be affected by a preceding general risk question. Consistent with this prediction, the mean of all specific questions was not different between the general‐first and specific‐first conditions, *F* (1, 1347) = 2.05, *p* = 0.153. H2 was supported.

H3 predicted an assimilation effect on the probability dimension when placed after the consequence dimension. However, there were no significant differences in probability dimension responses between the probability‐first and consequence‐first conditions, *F*(1, 1347) = 0.01, *p* = 0.920. H3 was not supported.

H4 predicted an assimilation effect on the consequence dimension when placed after the probability dimension. An order effect on the consequence dimension was observed, *F*(1, 1347) = 36.00, *p* < 0.001, 𝜂_p_^2^ = 0.026. As a whole, the consequence dimension of risk perception was reported as higher when it was asked first, *M* = 3.29, SD = 0.99, rather than when it was asked after probability dimensions, *M* = 2.98, SD = 1.05. This indicated that following exposure to probability dimensions, responses to consequence questions decreased, converging with the unconfounded responses to probability dimensions, *M* = 3.07, SD = 0.75, indicating an assimilation effect. Further supporting this assimilation trend, the correlation between the consequence and probability dimensions was greater in the probability‐first condition, as indicated by the positive interaction effect, *F*(1, 1345) = 12.54, *p* < 0.001, *η_p_* ^2^ = 0.009. Specifically, the coefficient of the probability dimension predicting the consequence dimension was 0.85 in the consequence‐first condition but increased to 1.01 in the probability‐first condition. H4 was supported.

RQ1 asked whether there were order effects between the two consequence subdimensions, severity and affect. Responses to the severity subdimension were significantly different between the severity‐first and affect‐first conditions, *F*(1,1347) = 9.29, *p* < 0.002, 𝜂_p_^2^ = 0.007. Specifically, the mean of the severity subdimension decreased following exposure to affect questions first, *M* = 3.42, SD = 1.15, compared to when severity questions were asked first, *M* = 3.58, SD = 1.15. Thus, the severity subdimension was influenced by preceding affect questions, converging with the unconfounded mean of the affect subdimension, *M* = 2.79, SD = 1.21, indicating an assimilation effect. However, this assimilation effect was not further supported in the moderation analysis—the severity‐affect order effect variable did not moderate the correlation between these two subdimensions, *F*(1, 1345) = 2.44, *p* = 0.119. Additionally, responses to the affect questions were unaffected by preceding severity questions, *F*(1, 1347) = 0.26, *p* = 0.609.

RQ2 concerned the order effects between the two probability subdimensions, exposure and susceptibility. Responses to the exposure subdimension were not affected by order effects, *F*(1, 1347) = 3.29, *p* = 0.070. Responses to the susceptibility subdimension significantly differed between the two order conditions, *F*(1, 1347) = 11.15, *p* < 0.001, 𝜂_p_^2^ = 0.008. Participants reported lower susceptibility when it was asked after the exposure questions, *M* = 3.26, SD = 1.12, relative to before, *M* = 3.47, SD = 1.08. Thus, after answering exposure questions first, susceptibility responses moved toward the unconfounded mean of the exposure block, *M* = 2.83, SD = 1.04, indicating an assimilation effect. Further supporting this trend, the moderation test revealed that the relationship between the exposure dimension and the susceptibility dimension became stronger when exposure questions were asked first, *F*(1, 1345) = 6.47, *p* = 0.011, *η_p_* ^2^ = 0.005. Specifically, the coefficient of exposure predicting susceptibility was 0.19 in the susceptibility‐first condition but increased to 0.33 in the exposure‐first condition.

**FIGURE 1:**
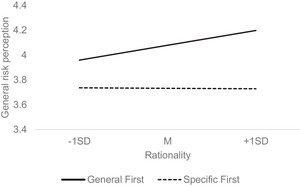
Simple slopes analysis showing how analytic thinking style moderates order effects in responses to the general risk perception question. Hazard type is included as a covariate in the analysis.

H5 predicted that order effects would be moderated by measures of cognitive sophistication—educational attainment, rationality, experientiality, and cognitive ability—such that individuals with greater levels of cognitive sophistication would be less prone to exhibit order effects. To each model that indicated a significant order effect above (H1, H4, RQ1, RQ2), we added each of these moderators in separate models. However, most of the cognitive sophistication variables did not moderate the effects observed in H1, H4, RQ1, and RQ2, all *p*s > 0.05. H5 was not supported. The only exception was that, contrary to our prediction, individuals higher in rationality were more likely to report lower general risk perception after first being exposed to specific questions than were those lower in rationality, *F*(1, 1345) = 5.69, *p* = 0.017, *η_p_* ^2^ = 0.004. Simple slopes analyses (Figure 1) indicated that general risk ratings were positively associated with rationality in the general‐first condition, but not in the specific‐first condition. In Figure 1, the wider gap between the two order conditions for high‐rationality individuals (+1SD), *b =* −0.47, SE = 0.07, *p* < 0.001, indicates that they were more sensitive to order effects than were low‐rationality individuals (−1SD), *b =* −0.22, SE = 0.07, *p* = 0.002.

## 5 Discussion

Although extensive order effects research has shown how responses to survey questions can be affected by their sequence, no research to our knowledge has tested their role in risk perception measurement. When different question orderings produce systematically different scores for the same underlying construct, this variation threatens construct validity by confounding true risk perception with contextual influences in measurement. The present study sought to identify the presence and types of order effects present across the dimensions in a recently developed risk perception scale. As expected, various order effects were found and suggest significant consequences related to survey question sequences as they are presented to participants.

### 5.1 Order Effects Between Risk Perception Dimensions

We found evidence of an assimilation effect when a general risk question followed specific items. This finding aligns with previously reported order effects of items in part‐whole relationships (Lee et al. 2023; Schwarz et al. 1991), suggesting that answering specific questions about the consequences and probability of a risk first creates a framework that shapes subsequent answers to the general risk question. The pattern is also consistent with research showing that the content of specific questions may function as anchors influencing subsequent judgments by making particular aspects of a concept more salient than others (Bless and Schwarz 2010; Kaplan et al. 2013). For instance, although people may globally judge tornadoes as highly risky based on mental images of devastating winds, considering first the actual probability of a tornado occurring in their community may lead them to incorporate this statistical perspective into subsequent general risk judgments, resulting in lower general risk responses. In contrast, answers to specific risk perception questions remained relatively stable, regardless of whether they were preceded by a general risk item. Overall, these findings suggest that when both general and specific risk perception questions are included in the questionnaire, asking the general question first can help obtain consistent responses relatively unaffected by question order.

Regarding order effects between probability and consequence dimensions, despite our prediction (H3), we found no evidence that probability dimensions were influenced by preceding consequence questions. However, supporting H4, we found evidence for assimilation effects influencing consequence questions following probability dimensions. This asymmetrical pattern suggests that thinking about the likelihood of exposure and susceptibility to a hazard may anchor and dampen the more affective judgments about consequences, but not vice versa. These findings are aligned with previous research suggesting that the valence of survey responses can be heavily influenced by accessible cognitions made salient while answering preceding items (Bowling and Windsor 2008; McClendon and O'Brien 1988; Moy et al. 2001). This finding also aligns with recent research showing that objective probability information reduces the dominance of consequence judgments in risk perception judgments (Hayakawa and Marian 2023). Given this asymmetry, it seems desirable to place consequence dimensions before probability dimensions in risk perception measurement to reduce order effects.

In our exploration of order effects between the two consequence subdimensions, we found partial evidence that the severity subdimension was influenced by preceding affect questions. In contrast, the affect subdimension was not influenced by preceding severity questions. Thus, it seems desirable to place the severity subdimension before the affect subdimension in questionnaires using multidimensional risk perception measurements.

Similarly, our analysis of order effects between the two probability subdimensions revealed that the susceptibility subdimension was influenced by preceding exposure questions, but not vice versa. We surmise that this unpredicted effect was observed likely due to the content of the two subdimensions where exposure may be construed as a precondition of susceptibility. Although exposure items measured community‐level likelihoods that a hazard will occur, susceptibility items asked how likely one is to experience the hazard's negative outcomes, assuming that exposure occurred. Although technically, such questioning requires a respondent to rate community‐level exposure and individual‐level susceptibility separately, our findings show that in practice, answering exposure items first further constrains participants’ judgments of susceptibility responses. For example, after answering that tornadoes are unlikely to happen in their community (i.e., exposure), they may further apply this exposure information to answer that their individual‐level threat (i.e., susceptibility) is low even when the question specifically asks to form a judgment assuming that a tornado occurred in their community. Given this asymmetric relationship, it would be preferred to place susceptibility or individual‐level questions before exposure or community‐level questions within the probability dimension to obtain responses relatively uninfluenced by question order effects.

Overall, these findings reveal an asymmetric pattern in question order effects in which questions requiring more analytic specificity—whether through consideration of specific risk features (specific questions), statistical assessment (probability), or community‐level occurrence (exposure)—tend to anchor evaluations that are more global (general risk), affective (consequence), or self‐focused (susceptibility) in nature. This pattern resulted in systematic measurement artifacts that researchers should account for in survey design.

### 5.2 Cognitive Sophistication and Order Effects

Contrary to our prediction that individuals with greater levels of cognitive sophistication (i.e., educational level, analytic thinking style, cognitive ability) would be less prone to order effects, we found no evidence supporting this hypothesis (H5) across order effects identified in our analyses. This suggests that question order effects in risk perception scales may not be driven by motivation or ability to engage with cognitive tasks. Instead, contrary to our predictions, we found that individuals higher in analytic thinking style (i.e., rationality) showed *stronger* order effects when answering general risk questions than did those lower in analytic thinking. As Figure 1 shows, while general risk ratings were higher in the general‐first condition than in the specific‐first condition across different levels of rationality, this tendency was particularly pronounced among high‐rationality individuals. This finding contradicts much of the existing literature, suggesting that higher cognitive sophistication should mitigate contextual influences in surveys (Lee et al. 2023; Narayan and Krosnick 1996). Instead, the finding aligns better with fuzzy‐trace theory, which suggests that experts in a domain rely more on fuzzy, intuitive representations of information (gist), than precise, detailed representations (verbatim), relative to novices (Reyna 2012). According to this perspective, individuals confident in their analytic thinking tendencies may engage more in global gist‐based processing, drawing on available summary judgments when rating the general risk of a hazard first. Yet, because high‐rationality individuals are also adept at processing probability information (Pacini and Epstein 1999), they actively adjusted their global risk judgment to more moderate levels when they considered specific aspects of the hazard first.

Theoretically, these findings challenge simplistic assumptions about the relationship between cognitive sophistication (and more specifically, analytic thinking) and susceptibility to question order effects. Further research is needed to replicate and fully examine this counterintuitive finding. Experimental studies that directly manipulate cognitive processing modes could help clarify the mechanisms underlying moderation effects. Replicating these effects in diverse contexts varying in risk level could further illuminate conditions under which cognitive sophistication moderates order effects in risk perception. For researchers and professionals using multidimensional scales to assess risk perception, it is important to note that general risk perception measures, when asked first as we suggest, can elicit divergent responses between individuals differing in analytic thinking propensity.

### 5.3 Limitations

A few limitations related to the study's external validity are noted. First, although the hazard contexts were not a focus of this study, all four were hazards that can impact whole communities as well as their individual constituents. Because parts of the scale assume community‐level impacts (e.g., exposure dimension), order effects may manifest differently when the scale is applied to hazards more isolated to an individual such as accidents, injuries, or specific diseases. Other multidimensional risk perception scales (e.g., Ferrer et al. 2016) may be relatively free from potential complications caused by assuming the presence of community‐level exposure. Second, in addition to sharing community‐level impacts, the four hazards tested were similar in nature in that they were natural or environmental hazards. Future research may test how the order effects observed in this study manifest across broader hazard categories including health, science, and technology. Lastly, our findings need to be interpreted with caution as we did not use a probabilistic sample. Although responses collected through MTurk tend to be diverse and reliable (Hauser and Schwarz 2016), especially when external quality filters are applied (Litman 2020), they cannot be considered representative of any population. Our participants were found to be leaning politically liberal, a characteristic that has been associated with elevated levels of risk perception (Dietz et al. 1998; Finucane, Slovic, et al. 2000). Similarly, our sample overrepresented individuals with high educational attainment, which could have affected our analyses testing the moderating influence of this variable on order effects.

## 6 Conclusion

This study demonstrates that question order systematically influences responses in multidimensional risk perception measurement, with asymmetrical patterns revealing that judgments requiring more analytic specificity anchored more global, affective, and self‐focused judgments when presented first. Our findings suggest that careful attention to the sequence of questions is necessary when designing risk perception instruments, as different dimensions vary in their susceptibility to contextual influences from preceding items. These findings offer practical direction for researchers and professionals seeking to minimize unintended measurement artifacts and enhance the validity of risk perception assessment across diverse hazards. Future scholars can extend this line of inquiry to further examine the underlying mechanisms involved in these question order effects and how these effects may vary depending on specific characteristics across hazard types.

## Conflicts of Interest

The authors declare no conflicts of interest.

## Funding

This study was funded by the Brian Lamb School of Communication, Purdue University.

## Supporting information




**Supporting Table S1**: Descriptive Statistics of Each Dimension by Question Order and Hazard type.
