# Supplementary material for: Question Order Effects in Multidimensional Risk Perception Measurement
Source: Risk Anal. 2025 Dec 16;46(1):e70164. doi: 10.1111/risa.70164 (PMC12857591; doi:10.1111/risa.70164)
Supplement: Supplementary file 1 — Supporting Table S1: Descriptive Statistics of Each Dimension by Question Order and Hazard type. [file RISA-46-0-s001.docx]

**Supplementary Material**

Table S1. *Descriptive Statistics of Each Dimension by Question Order and Hazard type*

| Hazard Type  / Dimension | Order: (A)-(B) | | | | | Order: (B)-(A) | | | | |
| --- | --- | --- | --- | --- | --- | --- | --- | --- | --- | --- |
|  | *n* | | *M* | *SD* | | *n* | | *M* | | *SD* |
| Poor Air Quality |  | |  |  | |  | |  | |  |
| General (A) | 171 | | 3.49^a^ | 1.06 | | 149 | | 3.17^b^ | | 1.05 |
| Specific (B) |  | | 2.91^a^ | 0.86 | |  | | 2.93^a^ | | 0.84 |
| Consequence (A) | 162 | | 3.11^a^ | 1.02 | | 158 | | 2.62^b^ | | 1.04 |
| Probability (B) |  | | 3.02^a^ | 0.84 | |  | | 2.91^a^ | | 0.76 |
| Severity (A) | 151 | | 2.91^a^ | 1.16 | | 169 | | 2.92^a^ | | 1.03 |
| Affect (B) |  | | 2.77^a^ | 1.20 | |  | | 2.86^a^ | | 1.17 |
| Susceptibility (A) | 147 | | 3.11^a^ | 1.01 | | 173 | | 2.96^a^ | | 1.14 |
| Exposure (B) |  | | 2.81^a^ | 0.87 | |  | | 3.00^a^ | | 0.93 |
| Heatwaves |  | |  |  | |  | |  | |  |
| General (A) | 169 | | 3.69^a^ | 1.05 | | 191 | | 3.47^b^ | | 1.02 |
| Specific (B) |  | | 3.04^a^ | 0.94 | |  | | 3.03^a^ | | 0.81 |
| Consequence (A) | 180 | | 2.96^a^ | 1.08 | | 180 | | 2.77^a^ | | 1.05 |
| Probability (B) |  | | 3.07^a^ | 0.87 | |  | | 3.22^a^ | | 0.81 |
| Severity (A) | 181 | | 3.16^a^ | 1.04 | | 179 | | 2.94^a^ | | 1.13 |
| Affect (B) |  | | 2.71^a^ | 1.19 | |  | | 2.66^a^ | | 1.21 |
| Susceptibility (A) | 181 | | 2.97^a^ | 1.10 | | 179 | | 2.88^a^ | | 1.04 |
| Exposure (B) |  | | 3.33^a^ | 0.97 | |  | | 3.42^a^ | | 0.96 |
| Tornadoes |  | |  |  | |  | |  | |  |
| General (A) | 171 | | 3.49^a^ | 1.06 | | 149 | | 3.17^b^ | | 1.05 |
| Specific (B) |  | | 2.91^a^ | 0.86 | |  | | 2.93^a^ | | 0.84 |
| Consequence (A) | 162 | | 3.11^a^ | 1.02 | | 158 | | 2.62^b^ | | 1.04 |
| Probability (B) |  | | 3.02^a^ | 0.84 | |  | | 2.91^a^ | | 0.76 |
| Severity (A) | 151 | | 2.91^a^ | 1.16 | | 169 | | 2.92^a^ | | 1.03 |
| Affect (B) |  | | 2.77^a^ | 1.20 | |  | | 2.86^a^ | | 1.17 |
| Susceptibility (A) | 147 | | 3.11^a^ | 1.01 | | 173 | | 2.96^a^ | | 1.14 |
| Exposure (B) |  | | 2.81^a^ | 0.87 | |  | | 3.00^a^ | | 0.93 |
| Lead Contamination | |  | | |  | |  | |  | |
| General (A) | 174 | | 4.43^a^ | 0.81 | | 161 | | 3.92^b^ | | 1.03 |
| Specific (B) |  | | 3.09^a^ | 0.79 | |  | | 3.02^a^ | | 0.74 |
| Consequence (A) | 161 | | 3.44^a^ | 0.85 | | 174 | | 3.14^b^ | | 1.01 |
| Probability (B) |  | | 2.95^a^ | 0.78 | |  | | 2.97^a^ | | 0.72 |
| Severity (A) | 163 | | 3.97^a^ | 0.94 | | 172 | | 3.79^a^ | | 0.98 |
| Affect (B) |  | | 2.63^a^ | 1.20 | |  | | 2.77^a^ | | 1.28 |
| Susceptibility (A) | 174 | | 3.81^a^ | 0.98 | | 161 | | 3.45^b^ | | 1.02 |
| Exposure (B) |  | | 2.27^a^ | 0.91 | |  | | 2.31^a^ | | 0.95 |

*Note.* In each pair of dimensions tested for order effects, one dimension is labeled (A) and the other (B). Means in the same row not sharing a superscript are significantly different from each other at *p* < .05 (two-sided) according to independent samples t-tests.
